# Synthesis and Electroluminescent Properties of Through-Space Charge Transfer Polymers Containing Acridan Donor and Triarylboron Acceptors

**DOI:** 10.3389/fchem.2019.00854

**Published:** 2019-12-10

**Authors:** Fan Chen, Jun Hu, Xingdong Wang, Shiyang Shao, Lixiang Wang, Xiabin Jing, Fosong Wang

**Affiliations:** ^1^State Key Laboratory of Polymer Physics and Chemistry, Changchun Institute of Applied Chemistry, Chinese Academy of Sciences, Changchun, China; ^2^School of Applied Chemistry and Engineering, University of Science and Technology of China, Hefei, China

**Keywords:** thermally activated delayed fluorescence, through-space charge transfer, triarylboron, electroluminescent polymer, organic light-emitting diodes

## Abstract

We report the design, synthesis and electroluminescent properties of three kinds of through-space charge transfer (TSCT) polymers consisting of non-conjugated polystyrene backbone, acridan donor and triarylboron acceptors having different substituents such as hydrogen (H), fluorine (F), and trifluoromethyl (CF_3_). Owing to the weak electron interaction between acridan donor and triarylboron acceptor through non-conjugated connection, blue emission with peaks in range of 429–483 nm can be achieved for the polymers in solid-state film, accompanied with photoluminescence quantum yields of 26–53%. The resulting TSCT polymers exhibit small ΔE_ST_ values below 0.1 eV owing to the separated HOMO and LUMO distributions, showing thermally activated delayed fluorescence with lifetimes in range of 0.19–0.98 μs. Meanwhile, the polymers show aggregation-induced emission (AIE) effect with the emission intensity increased by up to ~33 folds from solution to aggregation state. Solution-processed organic light-emitting diodes based on the polymers containing trifluoromethyl substituent exhibit promising electroluminescent performance with maximum luminous efficiency of 20.1 cd A^−1^ and maximum external quantum efficiency of 7.0%, indicating that they are good candidates for development of luminescent polymers.

## Introduction

Charge transfer (CT) is a crucial process in determining the emission behaviors of luminescent materials (Muller et al., 2003; Wu et al., 2004; Yuan et al., 2012; Liu et al., 2018; Sarma and Wong, 2018; Li J. et al., 2019). Luminescent polymers with CT emission have enabled important applications in solution-processed optoelectronic devices owing to their tunable emission color and promising luminescent efficiency (Yu et al., 2013; Bai et al., 2017). For example, thermally activated delayed fluorescence (TADF) polymers with finely manipulated CT process between electron donors and acceptors have emerged as attractive materials for organic light-emitting diodes (OLEDs) in recent years (Uoyama et al., 2012; Albrecht et al., 2015; Nikolaenko et al., 2015; Lee et al., 2016; Li et al., 2016, 2017; Luo et al., 2016; Zhu et al., 2016; Freeman et al., 2017; Wei et al., 2017; Wong and Zysman-Colman, 2017; Xie et al., 2017; Hu et al., 2018; Kim D. H. et al., 2018; Li C. S. et al., 2019). By separating the highest occupied molecular orbital (HOMO) and the lowest unoccupied molecular orbital (LUMO) orbitals to realize small singlet-triplet energy splitting (ΔE_ST_), the CT polymers with TADF characteristics can utilize the spin-forbidden triplet excitons through reverse intersystem crossing (RISC) process, shedding light on the potential of achieving solution-processed OLEDs with 100% internal quantum efficiency (IQE) based on pure organic polymers (Zhang et al., 2012; Hirata et al., 2015; Suzuki et al., 2015; Wang et al., 2015; Huang et al., 2018; Kim H. J. et al., 2018; Spuling et al., 2018; Wu et al., 2018; Ahn et al., 2019; Ban et al., 2019; Zhao et al., 2019).

So far, most luminescent polymers with CT emission are based on conjugated backbone, with feature of through-bond charge transfer (TBCT) emission from covalently bonded donors and acceptors. Owing to the strong electron coupling between donors and acceptors, the polymers are able to show large oscillator strength and high photoluminescence quantum efficiency (PLQY). However, the strong electron coupling mediated by covalent bonds tends to induce considerable red-shift of emission for the resulting polymers, undesirable for blue/deep blue emission. Meanwhile, ΔE_ST_ of the polymer can also be increased by the strong electron interaction between donor and acceptor, which could be unfavorable for realizing TADF effect (Li et al., 2016; Nobuyasu et al., 2016; Hu et al., 2018).

Different from conjugated donor-acceptor polymers with TBCT emission, non-conjugated polymers with through-space charge transfer (TSCT) emission between spatially separated acridan donors and triazine accepters have been reported to realize blue emission with TADF effect (Shao et al., 2017; Hu et al., 2019). Due to the physical separation of donor and acceptor, through-space charge transfer, rather than through-bond charge transfer occurs in this motif. This molecular design has the following merits. First, the non-conjugated polymer backbone avoids the strong electron coupling between donor and acceptor, favorable for blue emission of the resulting polymers. Second, the spatially separated donors and acceptors result in small overlap of HOMO and LUMO distributions, leading to small ΔE_ST_ and TADF effect. By modulating the CT strength through introducing substituents with different electron-accepting capability, TSCT polymers with emission color ranging from deep-blue to red can be realized with external quantum efficiency (EQE) up to 16.2%, suggesting their potential in development of novel luminescent materials for solution-processed OLEDs.

Recently triarylborons have been attractive building blocks for luminescent materials with CT character because of their promising electron-accepting properties endowed by the vacant p-orbitals of central boron atoms that can participate in π-conjugation with aryl groups (Hirai et al., 2015; Numata et al., 2015; Suzuki et al., 2015; Hatakeyama et al., 2016; Wu et al., 2018; Ahn et al., 2019; Kondo et al., 2019; Mellerup and Wang, 2019). The CT character of triarylboron-based donor–acceptor compounds strongly influences their photophysical properties and makes them useful for design of luminescent materials. For example, Adachi et al. first reported efficient blue TADF materials having a boron-containing acceptor combined with various donors, producing deep blue emission (450 nm) with maximum EQE of 20% (Numata et al., 2015). Recently, Hatakeyama et al. demonstrated triarylboron polycyclic aromatic compounds with multiple resonance effect of boron and nitrogen atoms, showing ultrapure blue emission with full-width at half-maximum of 18 nm and maximum EQE of 34.4%, indicating the great potential of triarylboron in developing efficient luminescent materials (Kondo et al., 2019).

Here we report the design, synthesis and properties of three kinds of through-space charge transfer polymers containing non-conjugated polystyrene backbone, acridan donor and triarylboron acceptors having different substituents such as hydrogen (H), fluorine (F), and trifluoromethyl (CF_3_). The triarylboron units are used as acceptors because of their weak electron-accepting capability which is favorable for realizing blue emission. By decorating the triarylboron acceptors with H, F, and CF_3_ groups to tune the charge transfer strength between donor and acceptor, the emission color can be tuned from deep blue (429 nm) to sky blue (483 nm) region in solid-state film, accompanied with improved photoluminescence quantum yield (PLQY) from 26 to 53%. The polymers exhibit small ΔE_ST_ values (<0.1 eV) because of the separated HOMO and LUMO distributions, allowing them to show TADF effect. Meanwhile, the polymers show aggregation-induced emission effect with the emission intensity increased by up to ~33 folds from solution to aggregation state (Luo et al., 2001; Hong et al., 2011; Mei et al., 2015). Solution-processed organic light-emitting diodes (OLEDs) based on the triarylboron-containing polymers show maximum EQEs up to 7.0%, indicating that they are promising candidates for development of luminescent polymers.

## Results and Discussion

### Molecular Design and Synthesis

**Figure 1:**
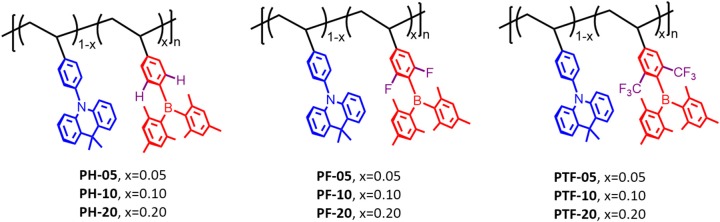
Molecular design and chemical structures of the through-space charge-transfer polymers containing triarylboron units.

To design luminescent polymers with through-space charge transfer, selection of polymer backbone, donor and acceptor plays the key role in determining the photophysical and electroluminescent properties of the resulting polymers. In this work polystyrene is selected as backbone because it provides the non-conjugated connection between donor and acceptor, while acridan is chosen as donor because of its good electron-donating ability as well as the rigid bridged structure. Moreover, triarylboron units are used as acceptors because they exhibit weak electron-accepting capability owing to the empty p_z_ orbital of boron that is capable to participate in π-conjugation with aryl groups. To tune the emissive color, three kinds of substituents hydrogen (H), fluorine (F), and trifluoromethyl (CF_3_) are introduced to the polymers, denoted as PH-05–PH-20, PF-05–PF-20, and PTF-05–PTF-20, respectively (Figure 1). The three substituents are selected because their electron affinity are gradually increased in order of H, F, and CF_3_, which can enhance the CT strength between the acridan donor and the triarylboron acceptor, allowing the modulation of TSCT emission of the resulting polymers. In addition, the content of acceptors are tuned at 5, 10, and 20 mol% to explore the influence of relative ratio between donor and acceptor on photophysical properties of the polymers.

**Scheme 1:**
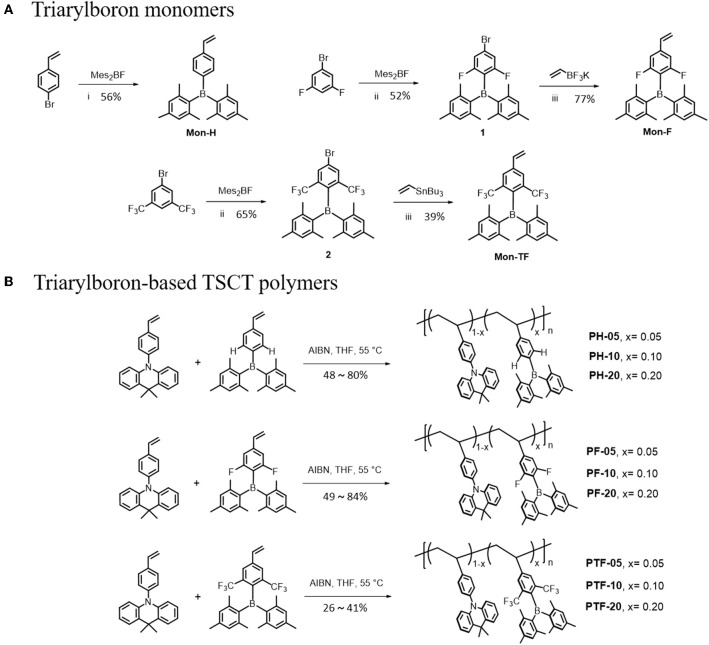
Synthetic routes for the triarylboron monomers **(A)** and TSCT polymers **(B)**. Reagents and conditions: (i) *n*-BuLi, THF, −78°C; (ii) LDA, THF, −78°C; (iii) Pd(PPh_3_)_2_Cl_2_, Cs_2_CO_3_, THF/H_2_O, 80°C, 24 h; (iv) Pd(PPh_3_)_4_, toluene, 105°C, 20 h.

Synthetic routes of the polymers are outlined in Scheme 1. The monomer Mon-H was prepared by a two-step procedure where the commercially available 4-bromovinylbenzene was first lithiated with *n*-BuLi, and then reacted with dimesityfluoroborane (Mes_2_BF) to afford the product in yield of 56%. For Mon-F and Mon-TF, 1-bromo-3,5-difluorobenzene (**1**) and 1-bromo-3,5-bis(trifluoromethyl)benzene (**3**) were first lithiated by lithium diisopropylamide (LDA) and then treated with Mes_2_BF to afford the bromide intermediates **2** and **4**. Consequently **2** and **4** were cross-coupled with vinyltrifluoroborate or tributylvinylstannane under palladium-catalyzed conditions to afford the desired monomers. With the monomers in hand, TSCT polymers were synthesized by free radical polymerization of the corresponding vinyl-functionalized acridan and triarylboron monomers using 2-azoisobutyronitrile as initiator and tetrahydrofuran (THF) as solvent. The content of triarylboron acceptors of the polymers are controlled through feed ratio (5–20 mol% for PX-5–PX-20). For comparison, three triarylboron model compounds bearing no vinyl groups and two control polymers with only acridan donors and triarylboron acceptors are also synthesized (Scheme S1). Number-average molecular weights (M_n_s) of the polymers measured by gel permeation chromatography using polystyrene as standard exhibit typical values of 9–45 KDa with polydispersity index (PDI) of 1.44–1.73 (Table 1). The decomposition temperatures (T_d_) of the polymers with 5% weight loss under nitrogen are higher than 300°C, while glass transition temperatures (T_g_) for the polymers are observed at 170–210°C (Figure S1). There are no exothermic peaks produced by crystallization within the scanning range, indicating the amorphous nature of the polymers. The TSCT polymers are readily soluble in common organic solvents, such as toluene, chloroform, tetrahydrofuran and chlorobenzene, ensuring the formation of high-quality films through solution process.

**Table 1:** Physical properties of the TSCT polymers.

**Polymer**	**M_n_^**a**^ (KDa)**	**PDI^**a**^**	**T_**g**_ (**°**C)**	**T_**d**_ (**°**C)**	**λ_PL_^**b**^ (nm)**	**λ_PL_^**c**^ (nm)**	**PLQY^**f**^ (%)**	**τ_**p**_/τ_**d**_^**d**^ (μs)**	**ΔE_**ST**_^**e**^ (eV)**
PH-05	21.8	1.68	176	356	438	429	26	0.007/0.19	0.076
PH-10	29.5	1.44	196	335	440	429	27	0.009/0.22	0.083
PH-20	43.2	1.55	193	303	439	435	27	0.008/0.21	0.096
PF-05	24.6	1.61	195	360	465	443	27	0.021/0.49	0.070
PF-10	31.6	1.51	197	343	467	453	30	0.036/0.44	0.073
PF-20	38.2	1.54	170	336	470	459	38	0.029/0.46	0.090
PTF-05	20.4	1.56	201	341	494	481	34	0.018/0.86	0.068
PTF-10	14.6	1.63	204	338	496	472	44	0.019/0.95	0.087
PTF-20	9.0	1.73	207	317	501	483	53	0.024/0.98	0.084

a *Determined by gel permeation chromatography with polystyrene standards*.

b *Measured in toluene at room temperature with a concentration of 1 × 10^−4^ M*.

c *Measured in neat films at room temperature*.

d *Lifetimes of prompt emission (τ_p_) and delayed emission (τ_d_) in toluene at 298 K in N_2_*.

e *Calculated from the onset wavelength of fluorescent and phosphorescent emission in film state*.

f *Absolute PL quantum yield in neat films determined in nitrogen*.

### Photophysical Properties

**Figure 2:**
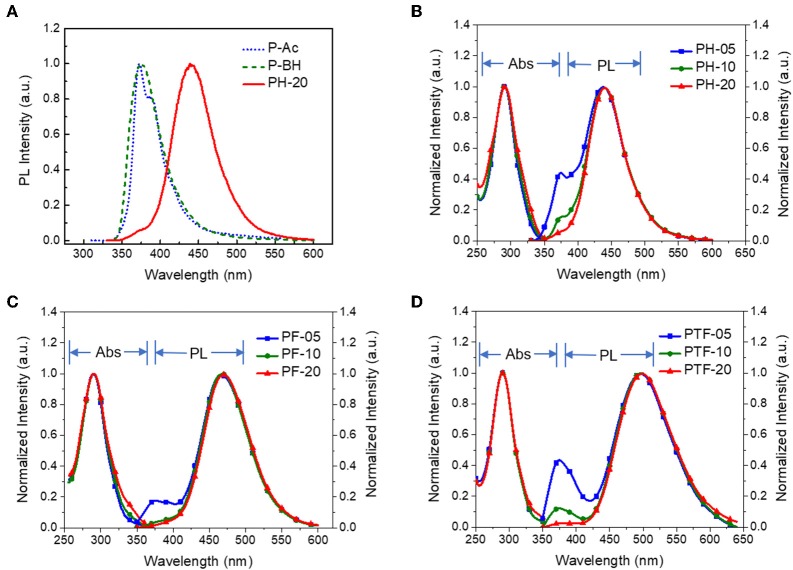
PL spectra of PH-20, P-Ac containing only acridan donor and P-BH containing only triarylboron acceptor **(A)**; as well as absorption and PL spectra of PH-05–PH-20 **(B)**, PF-05–PF-20 **(C)**, and PTF-05–PTF-20 **(D)** in toluene at 298 K with concentration of 1 × 10^−4^ M (λ_ex_ = 310 nm).

**Figure 3:**
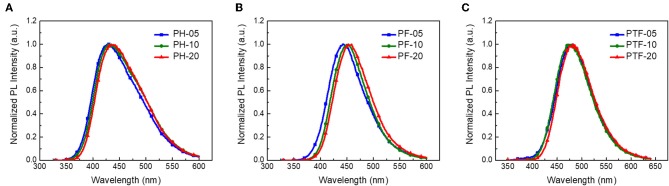
PL spectra of PH-05–PH-20 **(A)**, PF-05–PF-20 **(B)**, and PTF-05–PTF-20 **(C)** in film state.

The UV–vis absorption and fluorescence spectra of the polymers in toluene at 298K are shown in Figure 2, with the data summarized in Table 1. The polymers show similar absorption peaks at 290 nm which are mainly attributed to π-π^*^ transition of acridan and triarylboron units under diluted solutions. PL spectra of the polymers exhibit weak emission bands at 374 nm coming from the acridan unit, together with a strong, broad, and featureless emission bands at longer wavelength. These featureless bands are red-shifted compared with those of acridan and triarylboron units as well as the acridan- and triarylboron-containing homopolymers (Figure 2A and Figure S2). Moreover, these emissions show strong positive solvation effect as the polarity of the solvent increases. For instance, the emission maxima (λ_em, max_) of PH-20 shifts from 417 nm in cyclohexane to 476 nm in THF (Figure S3), confirming that the emissions are originating from CT transition between the donors and acceptors. By increasing electron-accepting ability of the triarylboron acceptors, the CT emission can be red-shifted from 438 nm (PH-05) to 465 nm (PF-05) and 494 nm (PTF-05). The content of the acceptor also have influence on the emission wavelength of the polymers. As the content of the acceptor increases from 5 to 20 mol%, the emission wavelength is red-shifted by 2–7 nm, consistent with the observations for triazine-based TSCT polymers (Shao et al., 2017). Different from those in solution, PL spectra of the polymers in film state show only CT emission from 429 to 483 nm (Figure 3), indicating that excited state energy of acridan donor has been completely transferred to the CT emissive species. The PLQYs of the polymers determined by integrating sphere is 26–27% for PH-05–PH-20, 27–38% for PF-05–PF-20 and 34–53% for PTF-05–PTF-20 (Table 1). As the content of acceptor increases, PLQYs of the polymers do not decrease, implying the weak concentration quenching effect of the TSCT emissive species.

**Figure 4:**
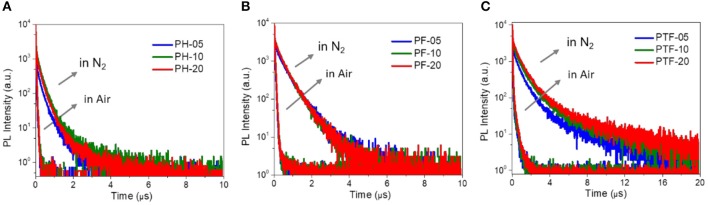
PL decay curves of PH-05–PH-20 **(A)**, PF-05–PF-20 **(B)**, and PTF-05–PTF-20 **(C)** in toluene under nitrogen/air at 298 K.

To investigate the TADF properties of the polymers, PL decay characteristics are measured in nitrogen and air. As shown in Figure 4 and Table 1, under nitrogen, all polymers in toluene displays distinctive delayed emissions with lifetimes (τ_d_) in microsecond scale, together with prompt emissions with lifetimes (τ_p_) in nanosecond scale. For example, PH-5, PF-5, and PTF-5 show τ_d_s of 0.19, 0.49, and 0.86 μs, respectively. The content of triarylboron units has slightly influence on τ_d_, with values of 0.21, 0.46, and 0.98 μs detected for PH-20, PF-20, and PTF-20, respectively. Under air, the delayed components are not detectable for all the polymers, indicating that the delayed emission is arising from triplets which can be quenched by oxygen, consistent with typical TADF behaviors. To further explore the TADF character, ΔE_ST_s of the polymers were determined from the onset of fluorescence spectra at room temperature and phosphorescence spectra at 77 K (Figure S4), which are smaller than 0.1 eV (Table 1). Such small ΔE_ST_s are consistent with the TADF effect since the rapid RISC process can be favored by small ΔE_ST_ to convert non-forbidden triplet excitons to radiative singlet excitons.

**Figure 5:**
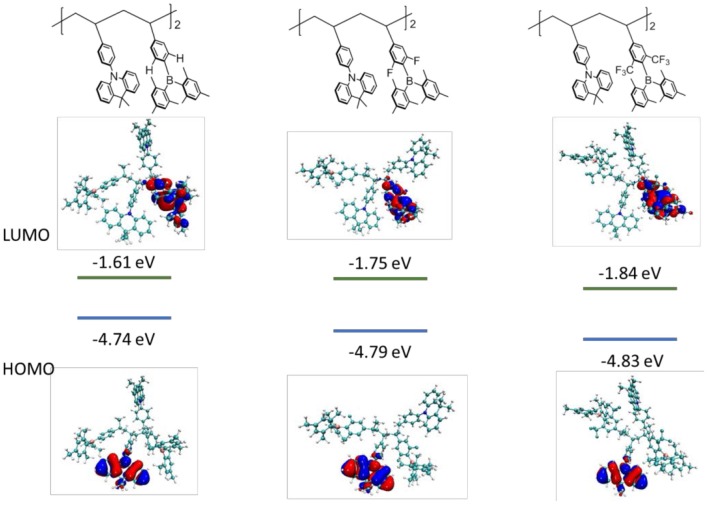
HOMO/LUMO distributions and energy levels for the polymer models consisting of two repeating units using density functional theory (DFT) method at B3LYP/6-31G(d) level.

To get insight into the electronic structures of the triarylboron-based TSCT polymers, frontier orbital distributions were investigated by density functional theory (DFT) calculations. It is found that for all the polymers with different substitution patterns, the HOMOs are predominantly located on the acridan units, whereas the LUMOs are distributed over the triarylboron acceptors, suggesting the CT character of the polymers (Figure 5). Moreover, the LUMO level of the polymers decreases from −1.61 to −1.75 eV and −1.84 eV as the substituent changes from H to F and CF_3_, indicating that the electron-accepting ability becomes stronger. Since the HOMO and LUMO are well-separated, the polymers show close singlet state (S_1_) and triplet state (T_1_) energy levels with the ΔE_ST_ values estimated to be ~zero according to time-dependent density functional theory (TD-DFT) calculations, which are consistent with the experimental ΔE_ST_ values.

**Figure 6:**
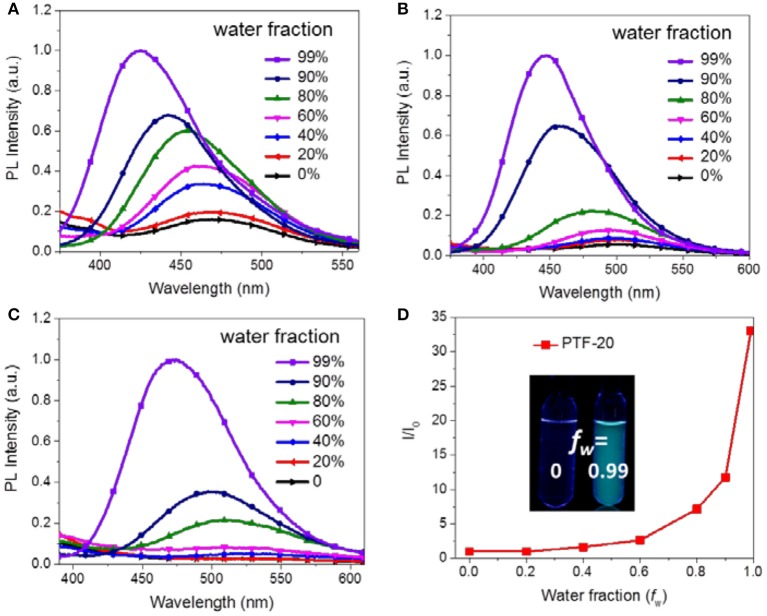
PL spectra of PH-20 **(A)**, PF-20 **(B)**, and PTF-20 **(C)** in THF/water mixture with different water fractions (concentration of polymers was 1 × 10^−5^ M, λ_ex_ = 310 nm), and relative emission intensity of PTF-20 in THF/water mixture **(D)**. Inset, PL images of polymers with different water fractions under 254 nm UV light.

It is noteworthy that the triarylboron-based TSCT polymers exhibit aggregation-induced emission (AIE) effect through measuring their PL spectra in THF/water mixed solvents (Luo et al., 2001; Hong et al., 2011; Mei et al., 2015). As shown in Figure 6, PTF-20 in pure THF solution shows a weak emission band at ~520 nm. As water is added, a slight increase in PL intensity was observed, which is accompanied with blue-shift of emission band. When the weight water (*f*_w_) is higher than 60%, the PL intensity increases drastically. Especially, at *f*_w_ of 99%, the polymer shows a dramatic increased intensity that is ~33 times higher than the initial THF solution (*f*_w_ = 0). Similar AIE effect is also observed for PH-20 and PF-20 with emission enhanced by ~6 and ~18 folds, respectively, as *f*_w_ increases from 0 to 0.99, which can be attributed to aggregation-induced dipole-dipole interaction between donors and acceptors.

### Electroluminescent Properties

**Figure 7:**
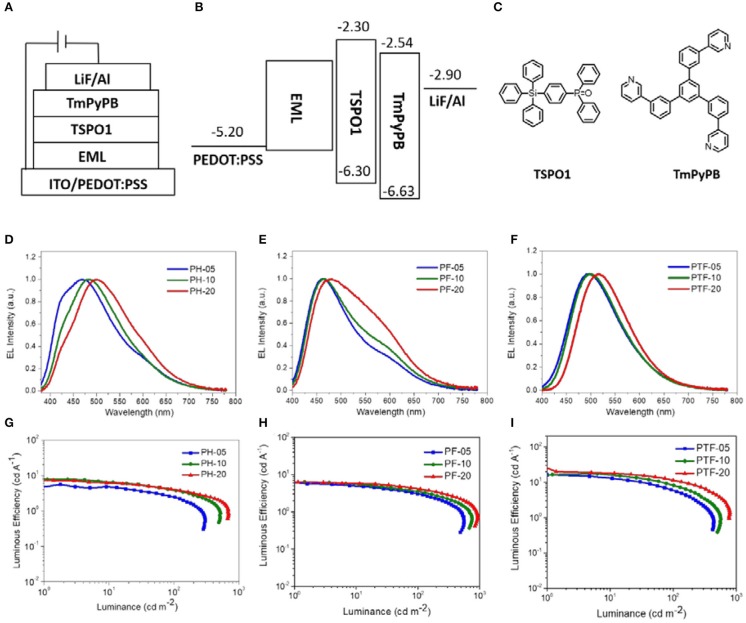
Device configuration **(A–C)**, EL spectra **(D–F)**, and current efficiency-luminance curves **(G–I)** of the solution-processed OLEDs.

To investigate the electroluminescent properties of the triarylboron TSCT polymers, solution-processed OLEDs were fabricated with device configuration of ITO/PEDOT:PSS (40 nm)/polymer (40 nm)/TSPO1 (8 nm)/TmPyPB(42 nm)/LiF (1 nm)/Al (100 nm) (Figure 7). Here PEDOT:PSS stands for poly(3,4-ethylenedioxythiophene):poly(styrene sulfonate) which serves as the hole-injection layer. TSPO1 (diphenyl(4-(triphenylsilyl)phenyl)phosphine oxide) (Mamada et al., 2011) and TmPyPB (1,3,5-tri(m-pyrid-3-yl-phenyl) benzene) (Su et al., 2008) act as the exciton blocking layer and the electron-transporting layer, respectively. As shown in Figures 7D–F, the polymers containing H substituents (PH-05, PH-10, and PH-20) show multiple emission bands at ~420 and ~480 nm. The former emission bands are similar to those observed in film-state PL spectra and therefore can be assigned to the CT emission. However, the origin of the latter emission bands is not clear yet. Similar behavior is also observed for PF-05–PF-20, showing CT emission at ~460 nm and unattributable emission bands at ~570 nm. Despite of this, the polymer bearing CF_3_ groups (PTF-05–PTF-20) show mainly CT emission regardless of the triarylboron content, with CIE coordinates in range of (0.25, 0.39)–(0.29, 0.47).

EQE–luminance and current density–voltage–luminance characteristics of the devices are shown in Figures 7G–I and Figure S5. The device performance is summarized in Table 2. All the devices show low driving voltages at 3.0–3.4 V, implying the good carrier injection and transport from the electrodes. The device efficiency of the TSCT polymers is dependant on both the substituent and the content of triarylboron units. For example, from PH-05 to PF-05 and PTF-05, the maximum LE increases from 5.5 to 16.3 cd A^−1^, and the maximum EQE increases from 2.9 to 5.7%. This observation is consistent with the enhanced PLQYs of the polymer films. Meanwhile, from PTF-05 to PTF-20 with increasing triarylboron content, the maximum LE increases slightly from 16.3 to 20.1 cd A^−1^, implying the negligible concentration quenching effect in the polymers. We note that the maximum EQEs of PTF-05–PTF-20 with values of 5.7–7.0% are much higher than the upper limit of conventional fluorescent materials (EQE = 5%), confirming the contributions of the triplets for EL emission, and indicating the promising potential of triarylboron TSCT polymers to serve as luminescent materials.

**Table 2:** Summary of the device performance of the TSCT polymers.

**Polymer**	**V_on_^**a**^ (V)**	**LE^**b**^ (cd/A)**	**EQE^**c**^ (%)**	**CIE (x, y)^**d**^**
		**Maximum value/at 100 cd m**^****−2****^		
PH-05	3.4	5.5/2.4	2.9/1.4	0.23, 0.27
PH-10	3.4	7.7/3.7	3.6/1.8	0.23, 0.31
PH-20	3.4	7.7/4.1	3.0/1.8	0.27, 0.36
PF-05	3.2	5.7/2.9	2.9/1.6	0.23, 0.26
PF-10	3.2	6.4/3.5	3.1/1.9	0.25, 0.29
PF-20	3.2	6.3/4.1	2.8/1.9	0.28, 0.34
PTF-05	3.0	16.3/6.2	5.7/2.6	0.25, 0.39
PTF-10	3.0	17.4/8.2	6.7/3.3	0.25, 0.41
PTF-20	3.0	20.1/11.4	7.0/4.2	0.29, 0.47

a *Turn-on voltage at the luminance of 1 cd m^−2^*.

b *Luminous efficiency*.

c *External quantum efficiency*.

d *CIE coordinates at 4 V*.

## Conclusion

In summary, three kinds of through-space charge transfer polymers with triarylboron acceptors bearing substituents of hydrogen (H), fluorine (F), and trifluoromethyl (CF_3_) are designed and synthesized for solution-processed OLEDs. The substitution effect on their photophysical and electroluminescent properties are investigated. It is found that as the substituent changes from H to F and CF_3_, the polymers show deep blue (429 nm) to sky-blue emission (483 nm) in solid-sate film due to the increase of electron-accepting ability of the triarylboron units. Owing to the small ΔE_ST_ of <0.1 eV, the TSCT polymers exhibit typical delayed fluorescence with τ_d_ of 0.19–0.98 μs in the absence of oxygen, with promising PLQY up to 53% in solid-state film. Aggregation-induced emission effect is observed for the polymers with the emission intensity increased by up to ~33 folds from solution to aggregation state. The TSCT polymer bearing trifluoromethyl substituent with 20 mol% acceptor content exhibits promising electroluminescent performance with maximum external quantum efficiency of 7.0%, suggesting that they are prospective candidates for the development of luminescent polymers in the future. Further investigation on enhancing the color purity and emission efficiency of the triarylboron-based TSCT polymers is currently underway.

## Data Availability Statement

The datasets generated for this study are available on request to the corresponding author.

## Author Contributions

FC did the polymer synthesis, photophysical measurements, and device investigations. JH and XW did the synthesis of monomers. FC and SS analyzed the photophysical and device data. SS and LW wrote the manuscript. LW, XJ, and FW organized the project.

### Conflict of Interest

The authors declare that the research was conducted in the absence of any commercial or financial relationships that could be construed as a potential conflict of interest.

## Supplementary Material

The Supporting Information includes the synthetic routes for model compounds and control polymers; TGA and DSC curves of the polymers; PL spectra of PH-20, PF-20, and PTF-20 in solutions with different polarities; Phosphorescence spectra of the TSCT polymers; Current density (J)–voltage (V)–luminance, and EQE–L characteristics of the solution-processed OLEDs based on TSCT polymers.
