# Supplementary material for: Synthesis and Electroluminescent Properties of Through-Space Charge Transfer Polymers Containing Acridan Donor and Triarylboron Acceptors
Source: Front Chem. 2019 Dec 10;7:854. doi: 10.3389/fchem.2019.00854 (PMC6914820; doi:10.3389/fchem.2019.00854)
Supplement: Supplementary file 1 [file Table_1.DOCX]

Supporting Information

**Synthesis and Electroluminescent Properties of Through-Space Charge Transfer Polymers Containing Acridan Donor and Triarylboron Acceptors**

*Fan Chen, Jun Hu, Xingdong Wang, Shiyang Shao,* Lixiang Wang,* Xiabin Jing, and Fosong Wang*

Table of Contents

- Experimental procedures
- Scheme S1. Synthetic routes for model compounds and control polymers
- Figure S1. Thermal properties of the TSCT polymers.
- Figure S2. Photophysical properties for model compounds of donor and acceptors
- Figure S3. PL spectra of the TSCT polymers in solutions with different polarities.
- Figure S4. Phosphorescence spectra of the TSCT polymers.
- Figure S5. J−V−L and EQE−L characteristics of the TSCT polymers.

**Experimental Procedures**

General information.

All chemicals and anhydrous solvents were purchased from commercial sources and we used without further purification unless stated otherwise. THF was distilled from sodium/benzophenone before use.

Measurement and characterization

NMR spectra were recorded on a Bruker Avance 400 NMR spectrometer. Chemical shifts are given in ppm, and are referenced against external Me_4_Si (^1^H, ^13^C), CFCl_3_ (^19^F) and BF_3_·OEt_2_ (^11^B). respectively. Elemental analyses were carried out using the Bio-Rad elemental analysis system. Number-average (M_n_) and weight-average (M_w_) molecular weights were determined against a polystyrene standard using an Waters 410 series GPC in THF at room temperature. Thermogravimetric analysis (TGA) was performed on a Perkin-Elmer-TGA 7, and differential scanning calorimetry (DSC) was measured on a PerkinElmer-DSC 7 instrument at a heating rate of 15 ^o^C/min under a N_2_ atmosphere. Cyclic Voltammetry experiments were performed on an EG&G 283 (Princeton Applied Research) potentiostat/galvanostat system using ferrocene as an internal reference and n-Bu_4_NClO_4_ as the supporting electrolyte. UV-visible absorption and Steady State photoluminescence spectra were recorded on a Perkin-Elmer Lambda 35 UV-vis spectrometer and a Perkin-Elmer LS 50B spectrofluorometer, respectively. The PLQY values were measured using an integrating sphere coupled with a photonic multichannel analyzer on Hamamatsu Photonics C9920‐02. PL attenuation curve was measured using picosecond pulsed diode laser under the excitation at 310 nm with Edinburgh fluorescence spectrometer (FLSP-980).

Fabrication and characterization of solution-processed organic light-emitting diode (OLEDs)

Glass substrates coated with indium tin oxide (ITO) layer with a sheet resistance of 15 Ω per square were cleansed using detergent, acetone, isopropanol and deionized water, which were then subjected to ultraviolet–ozone treatment for 25 minutes. A thin (≈30 nm) PEDOT:PSS (Clevious P AI4083) layer was spin‐coated on the cleaned ITO substrates at 5000 rpm for 60 s and was then baked at 120 ^o^C for 45 min in air then the substrate was transferred to a glove box with N_2_ atmosphere. The emitting layer was deposited by spin‐coating from a chlorobenzene solution containing 10 mg mL^−1^ of the TSCT polymers to form a film with thickness of 40 nm. The other layers including TSPO1 (8 nm)/TmPyPB (42 nm)/LiF (1 nm)/Al (100 nm) ware fabricated in a vacuum chamber at a base pressure of less than 4 × 10^-4^ Pa. The current density (J)–voltage (V)–luminance (L) characteristics were measured by a Keithley source measurement unit (Keithley 2400 and Keithley 2000) with a calibrated silicon photodiode under ambient atmosphere at room temperature. The electroluminescent spectra and CIE coordinates were measured by a PR650 spectra colorimeter.

**Scheme S1.** Synthetic routes for model compounds and control polymers


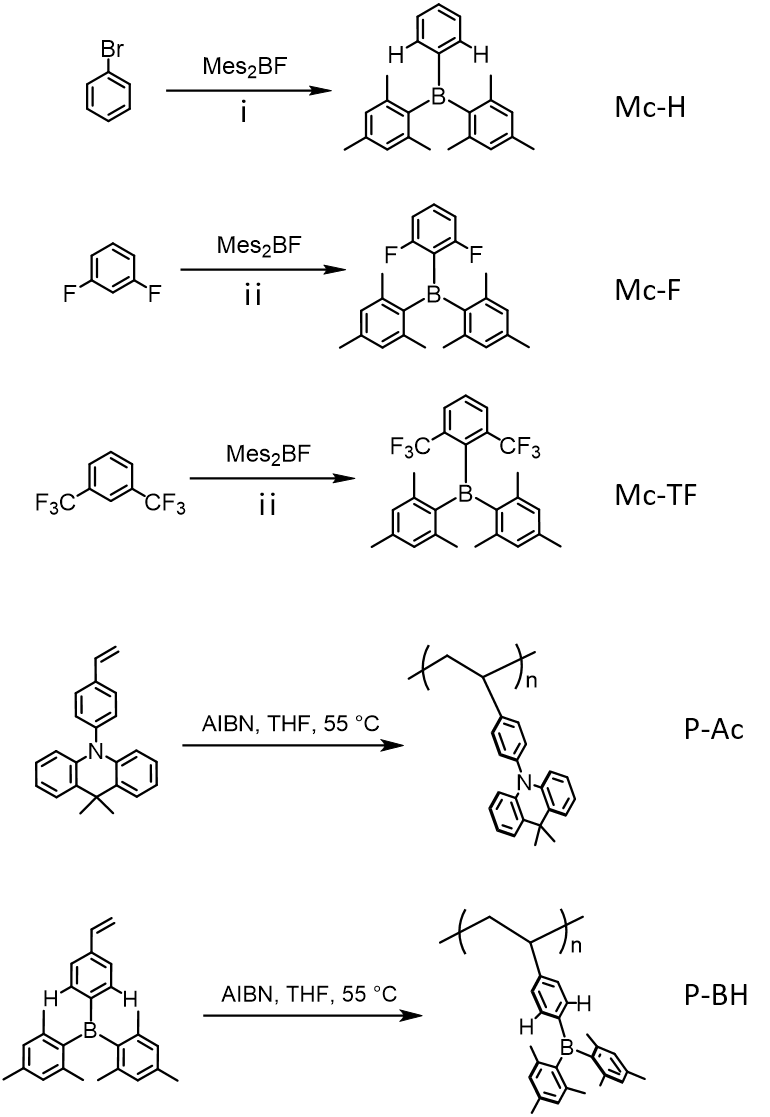


Reagents and conditions: (i) *n*-BuLi, THF, -78 ^o^C; (ii) LDA, THF, -78 ^o^C.


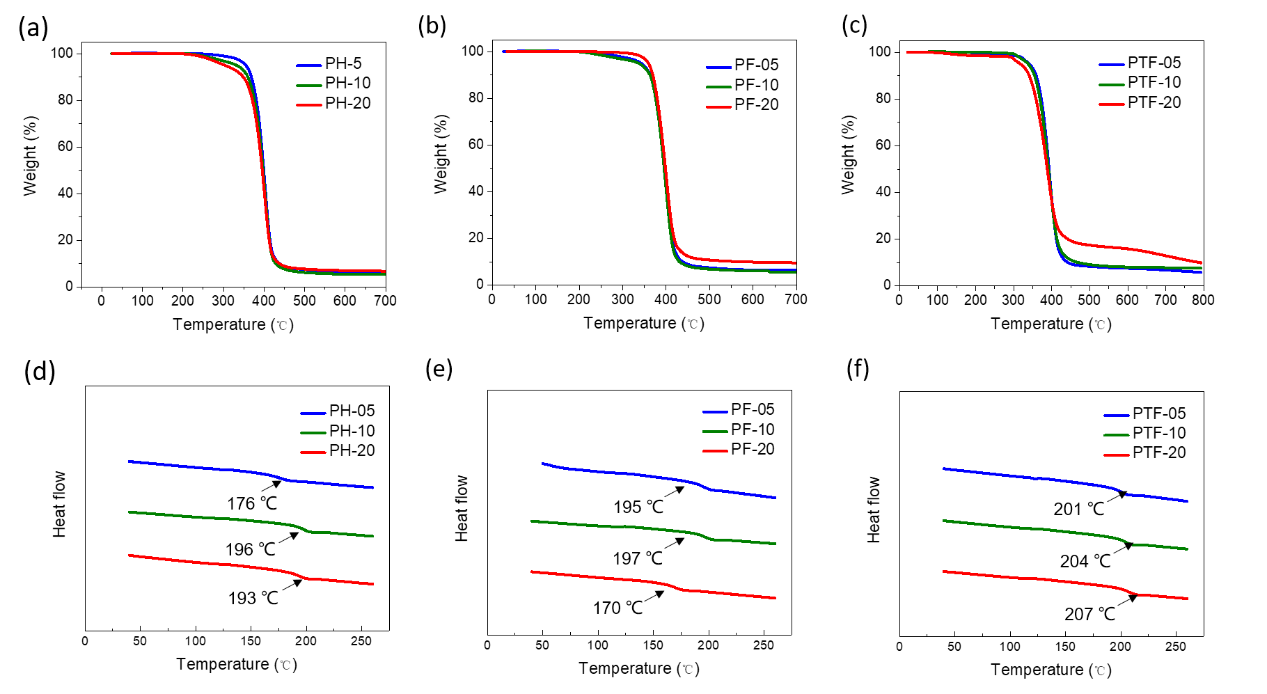
**Figure S1.** Thermogravimetic analysis (TGA) and differential scanning calorimetry (DSC) curves of the polymers.


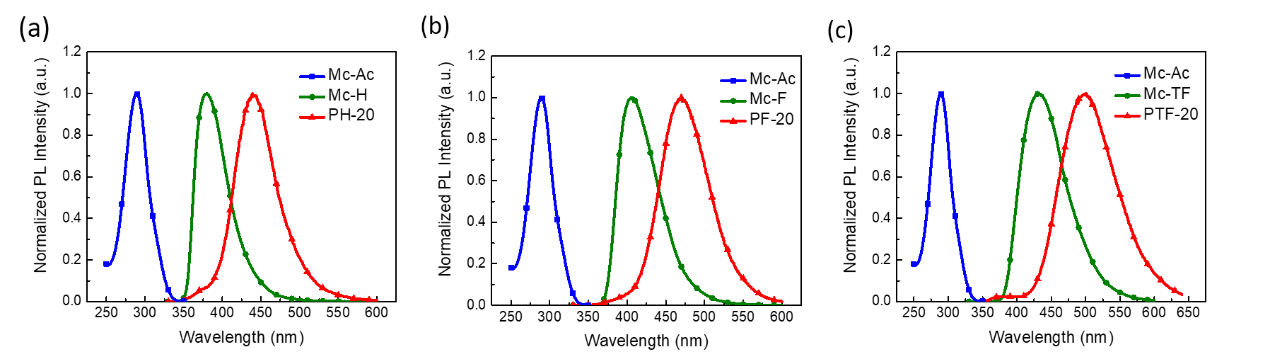
**Figure S2.** Photoluminescence (PL) spectra of the TSCT polymers and correspoding model compounds at room temperature in toluene at 10^-5^ mol L^-1^ (λ_ex_ = 290 nm)


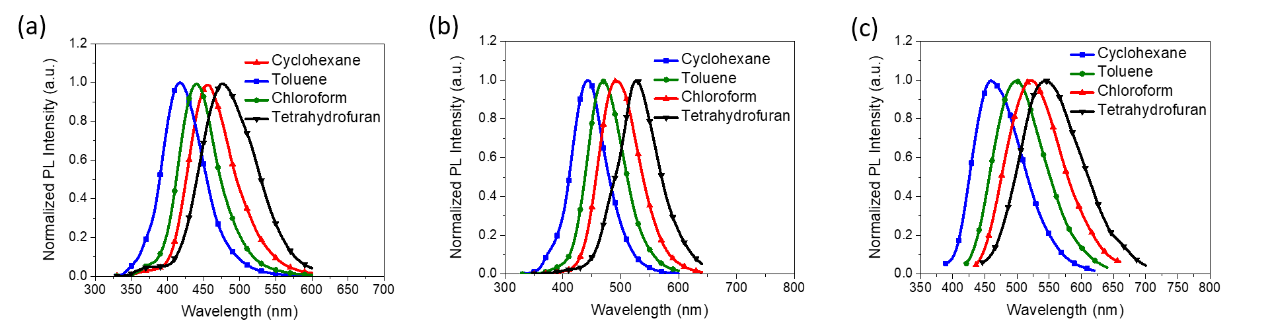
**Figure S3.** PL spectra of PH-20 (a), PF-20 (b) and PTF-20 (c) in solutions with different polarities.


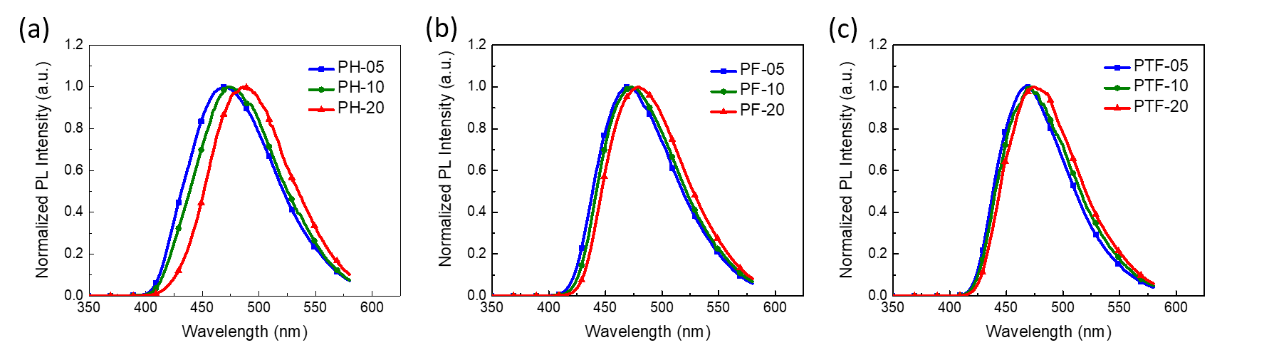
**Figure S4.** Phosphorescence spectra of the TSCT polymers in 77 K.


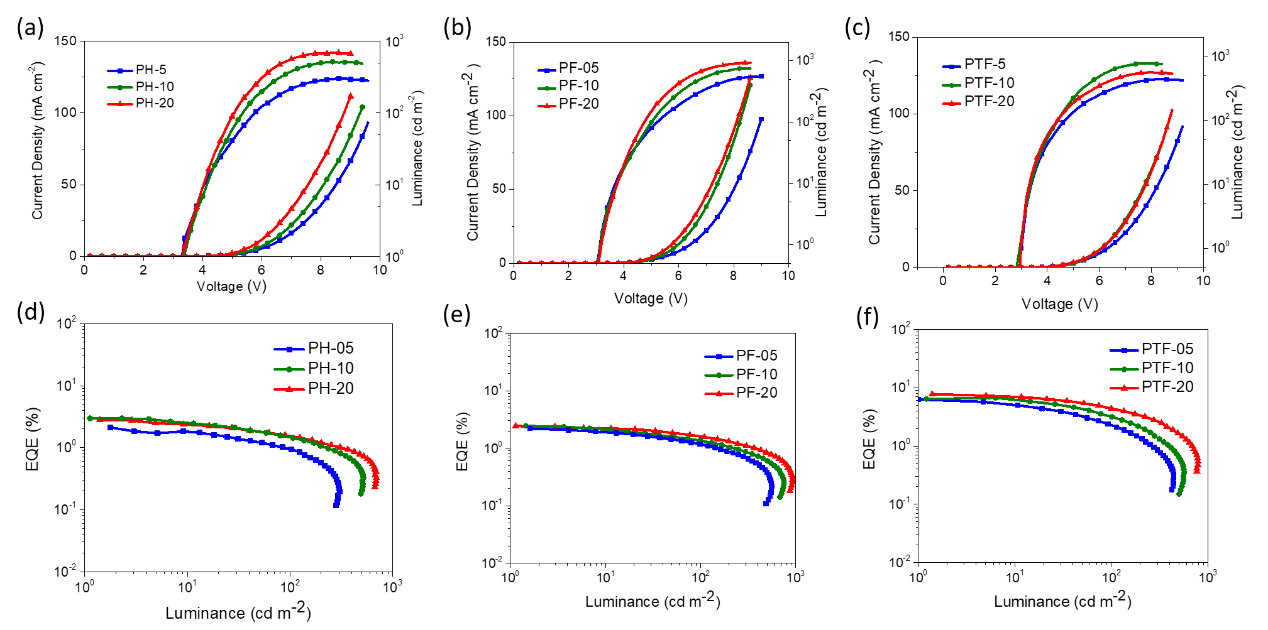


**Figure S5.** Current density (J)−voltage (V)−luminance (a-c) and EQE−L characteristics (d-f) of the solution-processed OLEDs based on TSCT polymers.

**Synthetic Procedures**

Dimesityl(4-vinylphenyl)borane (Mon-H)

A solution of 4-bromovinylbenzene (0.62 g, 3.4 mmol) in anhydrous tetrahydrofuran (40 mL) was cooled to -78 ^o^C and *n*-butyllithium 2.5 M in hexane (1.2 mL, 3.1 mmol) was added dropwise. The reaction mixture was stirred at -78 ^o^C for 2 hours. And then solution of dimesitylboron fluoride (1.00 g, 3.73 mmol) in tetrahydrofuran was slowly added to the mixture. The mixture was then warmed to room temperature and heated at 50 ^o^C for 2 hours. Subsequently, the reaction was cooled to room temperature and quenched with methanol (5 mL). After removal of the solvent under vacuum, water was added and the aqueous phase was extracted with dichloromethane. The combined organic fractions were dried over anhydrous Na_2_SO_4_, filtered and the solvent was removed under vacuum. The crude product was purified by silica gel chromatography using petroleum ether/dichloromethane (10/1) as eluent to give the product as a white solid (yield: 0.60 g, 56%). ^1^H NMR (500 MHz, CDCl_3_, δ): 7.49 (d, *J* = 8.0 Hz, 2H), 7.39 (d, *J* = 8.0 Hz, 2H), 6.83 (s, 4H), 6.75 (dd, *J* = 17.6, 10.9 Hz, 1H), 5.86 (d, *J* = 17.6 Hz, 1H), 5.33 (d, *J* = 11.0 Hz, 1H), 2.32 (s, 6H), 2.02 (s, 12H). ^13^C NMR (126 MHz, CDCl_3_, δ): 145.54, 141.86, 140.94, 138.72, 136.99, 136.97, 128.29, 125.93, 115.63, 23.59, 21.37.

(4-bromo-2,6-difluorophenyl)dimesitylborane (1)

[Lithium diisopropylamide](javascript:showMsgDetail('ProductSynonyms.aspx?CBNumber=CB6677718&postData3=CN&SYMBOL_Type=A');) (2 M, 1.6 mL, 3.2 mmol) was added dropwise to a stirred solution of 1-bromo-3,5-difluorobenzene (0.50 g, 2.6 mmol) in dry THF (100 mL) at −78 ^o^C. The mixture was stirred for 30 min, and then solution of dimesitylboron fluoride (0.76 g, 2.8 mmol) in tetrahydrofuran was slowly added to the mixture. The reaction mixture was slowly warmed to room temperature. After reaction, methanol and water was added successively and the aqueous phase was extracted with ether. The combined organic fractions were dried over anhydrous Na_2_SO_4_, filtered and the solvent was removed under vacuum. The crude product was purified by silica gel chromatography using petroleum ether as eluent. After removal of the solvents by evaporation, the product was obtained as a white solid (yield: 0.60 g, 52%). ^1^H NMR (400 MHz, CDCl_3_) δ 7.02 – 6.96 (m, 2H), 6.77 (s, 4H), 2.27 (s, 6H), 2.05 (s, 12H). ^11^B NMR (160 MHz, CDCl_3_): δ 75.62. ESI-MS: calcd for C_26_H_29_B: 352, found: 352 [M]+.

(2,6-difluoro-4-vinylphenyl)dimesitylborane (Mon-F)

A mixture of 1 (1.48 g, 3.4 mmol), potassium vinyltrifluoroborate (0.90 g, 6.7 mmol), bis(triphenylphosphine)palladium(II) chloride (Pd(PPh_3_)_2_Cl_2_) (0.05 g, 6.8 mmol), Cs_2_CO_3_ (2.20 g, 6.7 mmol) in THF (60 mL) and H_2_O (10 mL) was stirred at 85 ^o^C under argon for 22 h. After cooling to room temperature, the mixture was washed with water and the organic phase was dried with anhydrous Na_2_SO_4_. After removing the solvent, the mixture was applied to a silica gel column using petroleum ether/dichloromethane (50/1) as eluent to give the crude product Mon-F as white solid. (yield: 1.0 g, 77%). ^1^H NMR (500 MHz, CDCl_3_) δ 6.85 – 6.79 (m, 2H), 6.77 (s, 4H), 6.63 (dd, *J* = 17.5, 10.8 Hz, 1H), 5.80 (d, *J* = 17.5 Hz, 1H), 5.38 (d, *J* = 10.9 Hz, 1H), 2.27 (s, 6H), 2.07 (s, 12H). ^13^C NMR (126 MHz, CDCl_3_) δ 164.89 (d, *J* = 13.5 Hz), 162.93 (d, *J* = 13.4 Hz), 142.95 (t, *J* = 10.2 Hz), 141.94 (s), 140.23 (s), 139.59 (s), 135.03 (s), 128.41 (s), 116.88 (s), 109.02 – 108.19 (m), 22.68 (s), 21.30 (s). ^19^F NMR (471 MHz, CDCl_3_) δ -104.24 (s, 2F). ^11^B NMR (160 MHz, CDCl_3_): δ 75.82. ESI-MS: calcd for C_26_H_27_BF_2_: 388, found: 388 [M]+.

(4-bromo-2,6-bis(trifluoromethyl)phenyl)dimesitylborane (2)

[Lithium diisopropylamide](javascript:showMsgDetail('ProductSynonyms.aspx?CBNumber=CB6677718&postData3=CN&SYMBOL_Type=A');) (2 M, 4.1 mL, 8.2 mmol) was added dropwise to a stirred solution of [3,5-bis(trifluoromethyl)bromobenzene](javascript:showMsgDetail('ProductSynonyms.aspx?CBNumber=CB4137843&postData3=CN&SYMBOL_Type=A');) (2.00 g, 6.8 mmol) in dry THF (100 mL) at −78 ^o^C. The mixture was stirred for 30 min at −78 ^o^C and then solution of dimesitylboron fluoride (2.10 g, 7.5 mmol) in tetrahydrofuran was slowly added to the mixture. The reaction mixture was warmed to room temperature. After reaction, methanol and water was added successively and the aqueous phase was extracted with ether. The combined organic fractions where dried over anhydrous Na_2_SO_4_, filtered and the solvent was removed under vacuum. The crude product was purified by silica gel chromatography using petroleum ether as eluent. After removal of the solvents by evaporation, the product was obtained as a white solid (yield: 2.40 g, 65%).^1^H NMR (400 MHz, CDCl_3_) δ 7.90 (d, *J* = 33.1 Hz, 2H), 6.76 (dd, *J* = 11.4, 9.5 Hz, 4H), 2.27 (s, 6H), 1.94 (dd, *J* = 30.2, 19.2 Hz, 12H).

(2,6-bis(trifluoromethyl)-4-vinylphenyl)dimesitylborane (Mon-TF)

A mixture of 2 (1.00 g, 1.9 mmol), tributylvinylstannane (0.88 g, 2.8 mmol), Pd(PPh_3_)_4_ (0.11 g, 0.1 mmol) in toluene (20 mL) was stirred at 105 ^o^C under argon for 18 h. After cooling to room temperature, aqueous potassium fluoride solution was added. The mixture was filtered through celite and the residue was washed with dichloromethane. To the filtrate was added water and the mixture was extracted with dichloromethane. The organic layer was dried over anhydrous Na_2_SO_4_. After removing the solvent, the mixture was applied to a silica gel column using petroleum ether as eluent to give the crude product Mon-TF as white solid. (yield: 0.35 g, 39%). ^1^H NMR (500 MHz, CDCl_3_) δ 7.81 (s, 2H), 6.72 (m, 5H), 5.47 (dd, *J* = 17.1, 0.7 Hz, 1H), 5.09 (dd, *J* = 11.0, 0.6 Hz, 1H), 2.27 (d, *J* = 8.7 Hz, 6H), 1.97 – 1.87 (m, 9H), 1.74 (s, 3H). ^19^F NMR (471 MHz, CDCl_3_) δ -58.64 (s, 3F), -66.10 (s, 3F). ^11^B NMR (160 MHz, CDCl_3_): δ 75.90. ESI-MS: calcd for C_28_H_27_BF_6_: 488, found: 488 [M]^+^.

Dimesityl(phenyl)borane (Mc-H)

Mc-H was synthesized according to the same procedure as Mon-H. ^1^H NMR (500 MHz, CDCl_3_) δ 7.52 – 7.49 (m, 2H), 7.46 (dd, *J* = 10.4, 4.3 Hz, 1H), 7.33 (t, *J* = 7.5 Hz, 2H), 6.82 (s, 4H), 2.31 (s, 6H), 2.00 (s, 12H). ^13^C NMR (126 MHz, CDCl_3_) δ 145.83, 141.76, 140.81, 138.62, 136.32, 131.92, 128.15, 127.96, 23.40, 21.22. ^11^B NMR (160 MHz, CDCl_3_): δ 75.18. ESI-MS: calcd for C_24_H_27_B: 326, found: 326 [M]^+^.

(2,6-difluorophenyl)dimesitylborane (Mc-F)

[Lithium diisopropylamide](javascript:showMsgDetail('ProductSynonyms.aspx?CBNumber=CB6677718&postData3=CN&SYMBOL_Type=A');) (2 M, 1.55 mL, 3.1 mmol) was added dropwise to a stirred solution of 1,3-difluorobenzene (0.35 g, 3.1 mmol) in dry THF (25 mL) at −78 ^o^C. The mixture was stirred for 30 min at −78 ^o^C, and then a solution of dimesitylboron fluoride (0.83 g, 3.1 mmol) in tetrahydrofuran was slowly added to the mixture. After the reaction mixture was warmed to room temperature, methanol and water was added and the aqueous phase was extracted with ether. The combined organic fractions where dried over anhydrous Na_2_SO_4_, filtered and the solvent was removed under reduced pressure. The crude product was purified by silica gel chromatography using petroleum ether as eluent. After removal of the solvents by evaporation, the product was obtained as a white solid (yield: 0.55 g, 49%). ^1^H NMR (400 MHz, CDCl_3_) δ 7.39 – 7.28 (m, 1H), 6.82 – 6.73 (m, 6H), 2.27 (s, 6H), 2.07 (s, 12H). ^13^C NMR (126 MHz, CDCl_3_) δ 164.53 (d, *J* = 12.5 Hz), 162.56 (d, *J* = 12.5 Hz), 141.93 (s), 140.24 (s), 139.65 (s), 132.68 (t, *J* = 10.6 Hz), 128.43 (s), 111.20 – 110.86 (m), 22.61 (s), 21.30 (s). ^19^F NMR (471 MHz, CDCl_3_) δ 103.97 (s, 2F). ^11^B NMR (160 MHz, CDCl_3_): δ 76.32. ESI-MS: calcd for C_24_H_25_BF_2_: 362, found: 362 [M]^+^.

(2,6-bis(trifluoromethyl)phenyl)dimesitylborane (Mc-TF)

To a stirred solution of 1,3-bis(trifluoromethyl)benzene (0.50 g, 2.3 mmol) in dry THF (25 mL) was added dropwise [lithium diisopropylamide](javascript:showMsgDetail('ProductSynonyms.aspx?CBNumber=CB6677718&postData3=CN&SYMBOL_Type=A');) (2 M, 1.15 mL, 2.3 mmol) at −78 ^o^C. The mixture was stirred for 30 min at that temperature, and then dry tetrahydrofuran solution of dimesitylboron fluoride (0.63 g, 2.3 mmol) was slowly added to the mixture. The reaction mixture was gradually warmed to room temperature. After addition of methanol, water was added and the aqueous phase was extracted with ether. The combined organic fractions where dried over anhydrous Na_2_SO_4_, filtered and the solvent was removed under reduced pressure. The crude product was purified by silica gel chromatography using petroleum ether as eluent. After removal of the solvents by evaporation, the resulting solid Mc-F was as a white solid (yield = 0.18 g, 17%). ^1^H NMR (400 MHz, CDCl_3_) δ 7.91 (s, 1H), 7.71 (d, *J* = 8.0 Hz, 1H), 7.47 (d, *J* = 7.9 Hz, 1H), 6.77 (s, 4H), 2.30 (d, *J* = 16.6 Hz, 6H), 1.92 (d, *J* = 17.6 Hz, 12H). ^19^F NMR (471 MHz, CDCl_3_) δ -60.08 (s, 3F), -66.04 (s, 3F). ^11^B NMR (160 MHz, CDCl_3_): δ 76.82. ESI-MS: calcd for C_26_H_25_BF_6_: 462, found: 462 [M]^+^.

General polymerization procedure for the polymers.

The polymers were synthesized by free radical polymerization in THF at 55 ^o^C. THF was freshly distilled to remove the stabilizer. Azodiisobutyronitrile (AIBN) was used as radical initiator and was crystallized from diethyl ether before use. For polymerization, AIBN (2 mol% relative to the total amount of the monomers) and the monomers were dissolved in THF, and then stirred at 55 ^o^C for 48 h under argon. After cooling to room temperature, the solution was precipitated into methanol twice. The mixture was filtered and dried under vacuum to give the resultant polymer as a white fiber in yield of 26–84%.

PH-05. White fiber (0.20 g). Yield: 48%. M-Ac (0.40 g, 1.28 mmol) and Mon-H (0.024 g, 0.068 mmol) were used in the polymerization. ^1^H NMR (500 MHz, CDCl_3_) δ 7.7-7.2 (aromatics), 7.2-5.9 (aromatics), 2.4-1.2 (backbone). ^13^C NMR (126 MHz, CDCl_3_) δ 140.78, 137.88, 131.43, 129.99, 129.04, 128.23, 126.25, 125.30, 125.09, 120.62, 113.57, 35.81, 31.01, 21.46.

PH-10. White fiber (0.23 g). Yield: 50%. M-Ac (0.40 g, 1.28 mmol) and Mon-H (0.050 g, 0.143 mmol) were used in the polymerization. ^1^H NMR (500 MHz, CDCl_3_) δ 7.7-7.2 (aromatics), 7.2-5.9 (aromatics), 2.4-1.2 (backbone). ^13^C NMR (126 MHz, CDCl_3_) δ 140.86, 131.52, 129.05, 128.23, 126.32, 125.31, 120.67, 120.54, 114.01, 35.82, 30.91.

PH-20. White fiber (0.36 g). Yield: 80%. M-Ac (0.35 g, 1.12 mmol) and Mon-H (0.099 g, 0.281 mmol) were used in the polymerization. ^1^H NMR (500 MHz, CDCl_3_) δ 7.7-7.2 (aromatics), 7.2-5.9 (aromatics), 2.4-1.2 (backbone). ^13^C NMR (126 MHz, CDCl_3_) δ 140.86, 137.93, 131.46, 129.94, 129.05, 128.24, 126.36, 125.31, 120.53, 114.01, 35.81, 31.29, 21.46.

PF-05. White fiber (0.22 g). Yield: 52%. M-Ac (0.40 g, 1.28 mmol) and Mon-F (0.026 g, 0.068 mmol) were used in the polymerization. ^1^H NMR (500 MHz, CDCl_3_) δ 7.7-7.2 (aromatics), 7.2-5.9 (aromatics), 2.4-1.2 (backbone). ^13^C NMR (126 MHz, CDCl_3_) δ 140.73, 139.49, 131.40, 129.97, 129.03, 128.58, 128.22, 126.34, 125.21, 120.52, 114.01, 113.55, 35.80, 31.24, 31.05, 22.71, 21.45. ^19^F NMR (471 MHz, CDCl_3_) δ -102.74.

PF-10. White fiber (0.22 g). Yield: 49%. M-Ac (0.40 g, 1.28 mmol) and Mon-F (0.055 g, 0.143 mmol) were used in the polymerization. ^1^H NMR (500 MHz, CDCl_3_) δ 7.7-7.2 (aromatics), 7.2-5.9 (aromatics),2.4-1.2 (backbone). ^13^C NMR (126 MHz, CDCl_3_) δ 140.74, 139.54, 136.06, 131.40, 130.01, 129.04, 128.58, 128.23, 126.34, 125.21, 120.52, 114.01, 113.54, 35.82, 31.26, 31.01, 22.70, 21.46. ^19^F NMR (471 MHz, CDCl_3_) δ -102.71.

PF-20. White fiber (0.44 g). Yield: 84%. M-Ac (0.40 g, 1.28 mmol) and Mon-F (0.125 g, 0.321 mmol) were used in the polymerization. ^1^H NMR (500 MHz, CDCl_3_) δ 7.7-7.2 (aromatics), 7.2-5.9 (aromatics),2.4-1.2 (backbone). ^13^C NMR (126 MHz, CDCl_3_) δ 140.73, 139.91, 139.54, 136.06, 131.43, 129.97, 129.03, 128.58, 128.22, 126.34, 125.30, 125.12, 120.52, 114.92, 114.04, 113.60, 35.82, 31.26, 31.06, 22.67, 21.45, 21.15. ^19^F NMR (471 MHz, CDCl_3_) δ -102.65.

PTF-05. White fiber (0.11 g). Yield: 26%. M-Ac (0.40 g, 1.28 mmol) and Mon-TF (0.033 g, 0.068 mmol) were used in the polymerization. ^1^H NMR (500 MHz, CDCl_3_) δ 7.2-5.8 (aromatics), 2.4-1.2 (backbone). ^13^C NMR (126 MHz, CDCl_3_) δ 140.73, 139.50, 137.87, 131.40, 129.93, 129.03, 128.22, 126.22, 125.33, 125.08, 120.53, 113.52, 35.81, 31.05, 21.45. ^19^F NMR (471 MHz, CDCl_3_) δ -57.64, -58.62, -65.48, -66.08.

PTF-10. White fiber (0.19 g). Yield: 41%. M-Ac (0.40 g, 1.28 mmol) and Mon-TF (0.070 g, 0.143 mmol) were used in the polymerization. ^1^H NMR (500 MHz, CDCl_3_) δ 7.2-5.8 (aromatics), 2.4-1.2 (backbone). ^13^C NMR (126 MHz, CDCl_3_) δ 140.73, 139.52, 137.87, 131.28, 129.95, 129.04, 128.23, 126.19, 125.30, 125.06, 120.62, 113.57, 35.80, 31.05, 21.46. ^19^F NMR (471 MHz, CDCl_3_) δ -57.66, -58.62, -65.48, -66.09.

PTF-20. White fiber (0.22 g). Yield: 40%. M-Ac (0.40 g, 1.28 mmol) and Mon-TF (0.157 g, 0.321 mmol) were used in the polymerization. ^1^H NMR (500 MHz, CDCl_3_) δ 7.2-5.8 (aromatics), 2.4-1.0 (backbone). ^13^C NMR (126 MHz, CDCl_3_) δ 140.72, 131.31, 129.95, 129.03, 128.22, 126.19, 125.11, 120.52, 113.52, 35.81, 31.01, 21.45. ^19^F NMR (471 MHz, CDCl_3_) δ -57.67, -65.48.

P-Ac. White fiber (0.30 g). Yield: 75%. Mon-Ac (0.40 g, 1.28 mmol) were used in the polymerization. ^1^H NMR (500 MHz, CDCl_3_) δ 7.2-7.0 (aromatics), 6.9-6.0 (aromatics), 2.7-1.7 (backbone), 1.7-1.3 (backbone). ^13^C NMR (126 MHz, CDCl_3_) δ 140.74, 139.48, 131.40, 129.97, 128.58, 126.34, 126.22, 125.21, 125.12, 120.52, 114.01, 113.53, 40.97, 35.80, 31.26, 31.05.

P-BH. White fiber (0.30 g). Yield: 75%. Mon-H (0.40 g, 1.14 mmol) were used in the polymerization. ^1^H NMR (500 MHz, CDCl_3_) δ 7.2-7.0 (aromatics), 6.9-6.1 (aromatics), 2.4-2.0 (backbone), 2.0-1.6 (backbone). ^13^C NMR (126 MHz, CDCl_3_) δ 141.66, 140.51, 138.22, 136.46, 129.04, 128.23, 128.13, 125.30, 23.36, 21.46, 21.22.
